# Differential response to gefitinib of cells expressing normal EGFR and the mutant EGFRvIII

**DOI:** 10.1038/sj.bjc.6602793

**Published:** 2005-09-27

**Authors:** M W Pedersen, N Pedersen, L H Ottesen, H S Poulsen

**Affiliations:** 1 Department of Radiation Biology, The Finsen Center, Copenhagen University Hospital, Blegdamsvej 9, Copenhagen DK-2100, Denmark; 2 Medical Department, AstraZeneca, Roskildevej 22, Albertslund DK-2620, Denmark

**Keywords:** EGFR, EGFRvIII, iressa, gefitinib, signalling, mutation

## Abstract

Epidermal growth factor receptor (EGFR) is frequently amplified and/or mutated in a number of human tumours and abnormal signalling from this receptor is believed to contribute to the malignant phenotype seen in these tumours. Gefitinib is a small molecule inhibitor that specifically binds and inhibits the EGFR tyrosine kinase and has been shown to inhibit the growth, proliferation, survival and invasion of a range of tumour cells overexpressing EGFR. However, clinical response to gefitinib has failed to correlate with EGFR levels and activity, indicating that other molecular mechanisms such as downstream signalling and mutations could be of importance in predicting clinical response. We therefore investigated the effect of the specific EGFR inhibitor gefitinib on the phosphorylation level, signalling and growth of cells expressing the naturally occurring constitutively active EGFR variant EGFRvIII, a low nontransforming level of EGFR and a high transforming level of EGFR. Results show that levels of gefitinib sufficient to suppress EGFR phosphorylations, EGFR-mediated proliferation and EGFR-mediated anchorage-independent growth are not sufficient to inhibit these features in cells expressing EGFRvIII. Furthermore, the data indicate that long-term exposure of EGFRvIII-expressing cells to low concentrations of gefitinib (0.01–0.1 *μ*M) result in increased phosphotyrosine load of the receptor, increased signalling to ERK and stimulation of proliferation and anchorage-independent growth, presumably by inducing EGFRvIII dimerisation. Higher concentrations of gefitinib (1–2 *μ*M), on the other hand, significantly decreased EGFRvIII phosphotyrosine load, EGFRvIII-mediated proliferation and anchorage-independent growth. Further studies are needed to investigate the implications of these important findings in the clinical setting.

Receptor tyrosine kinases regulate signalling pathways involved in critical cellular activities such as growth, proliferation, motility, survival and apoptosis. When activated by overexpression, autocrine growth factor stimulation or mutations, receptor tyrosine kinases can contribute to the development of human cancers. The epidermal growth factor receptor (EGFR, ErbB1) is a tyrosine kinase receptor of the ErbB family and is frequently overexpressed or mutated in human malignancies including those of the brain, breast, colon, ovary and lung (Tang *et al*, 2000; Alper *et al*, 2001; Ge *et al*, 2002; Peghini *et al*, 2002). This has inspired the development of specific pharmacological inhibitors of the EGFR tyrosine kinase such as gefitinib (Iressa, AstraZeneca Pharmaceuticals), which disrupts EGFR kinase activity by reversibly binding within the ATP-binding pocket of the EGFR protein (Arteaga and Johnson, 2001). Gefitinib is orally active and has shown promising antitumour activity *in vitro* and *in vivo*, although clinical response has failed to correlate with either EGFR levels or activity. Recently, however mutations were identified in the tyrosine kinase domain, which were associated with sensitivity of non-small-cell lung cancer to gefitinib (Lynch *et al*, 2004; Paez *et al*, 2004).

The type III epidermal growth factor mutation variously named, EGFRvIII, ΔEGFR, de2-7EGFR and mEGFR is the most common mutation in the EGFR gene and is frequently found in tumours of the breast, ovary, prostate, lung and in particular those of the brain (Garcia *et al*, 1993; Moscatello *et al*, 1995; Olapade-Olaopa *et al*, 2000; Pedersen *et al*, 2001; Ge *et al*, 2002). This mutation or deletion eliminates exons 2–7 resulting in a truncated receptor with a distorted ligand-binding area. However, despite lack of ligand-binding EGFRvIII has a constitutively active receptor tyrosine kinase and is able to transform fibroblasts and to confer enhanced tumorigenicity to cancer cells both *in vitro* and *in vivo* (Huang *et al*, 1997; Damstrup *et al*, 2002; Pedersen *et al*, 2004). A recent study found that cells expressing EGFRvIII had increased resistance to gefitinib *in vitro* and *in vivo*, which were attributed to a deficiency in receptor dephosphorylation and constitutive AKT activity (Learn *et al*, 2004). Hence, evidence accumulates that signalling regulated by EGFR is altered by overexpression or mutations and that such alterations can lead to differences in response to gefitinib treatment.

The purpose of this study was to investigate the effect of gefitinib on phosphorylation level, intracellular signalling and growth properties of cells expressing EGFRvIII. However, there are several complicating factors associated with the analysis of EGFRvIII-mediated signalling in cancer cells. In particular, the enhanced tumorigenicity conferred to cancer cells by EGFRvIII is predominantly restricted to *in vivo* growth conditions making detailed analyses problematic. Many cancer cells also express significant levels of other members of the ErbB family that are potentially capable of forming heterodimers with EGFRvIII (O'Rourke *et al*, 1998).

Therefore we have used the unique NR6 model system, in which NR6wtEGFR express a low nontransforming level of EGFR and the fully tumorigenic cell lines NR6W and NR6M that overexpress EGFR or express mutant EGFRvIII, respectively (Batra *et al*, 1995). This allows us to characterise the effect of gefitinib on phosphorylation and intracellular signalling by a nontransforming level of EGFR, by a transforming level of EGFR and by a transforming level of EGFRvIII.

Results show that some tyrosine phosphorylation sites on the wild-type (wt)-EGFR are more resistant to inhibition by gefitinib compared to others. Results also confirm that higher doses of gefitinib are needed to abrogate phosphorylation of and signalling to PLC-*γ* and AKT by EGFRvIII as compared to wt-EGFR. Furthermore, small levels of gefitinib seem to induce receptor autophosphorylation, as well as proliferation and anchorage-independent growth of EGFRvIII-expressing cells.

## MATERIALS AND METHODS

### Materials

Recombinant human EGF was purchased from Calbiochem (Germany). Anti EGFR, phospho-EGFR (Tyr845, Tyr992, Tyr1045, Tyr1068), STAT3, phospho-STAT3 (Tyr705), AKT, phospho-AKT (Ser473), extracellular-regulated kinase (ERK)-1/2 and phospho-ERK-1/2 (Thr202/Tyr204) antibodies were from Cell Signaling Technology (Germany). Antiphospho-EGFR (Tyr1148 and Tyr1173) antibodies were from Upstate Biotechnology (USA). The antiphospho-EGFR (Tyr1086) antibody was from Biosource (USA).

Antibody to tubulin was from Santa Cruz Biotechnology Inc. (CA, USA). HRP-conjugated secondary antibodies were purchased from, DAKO (Denmark).

### Cell lines

Four cell lines were included in this work. The cell lines NR6, NR6M and NR6W have been described previously and were kindly provided by Dr Darell Bigner, Duke University, NC, USA (Batra *et al*, 1995). The NR6wtEGFR cell line, which expresses a lower number of receptors as compared to NR6W, has also been described previously and is a generous gift from Dr Allan Wells, Department of Pathology, University of Pittsburgh (Wells *et al*, 1990).

### Immunoblot analyses

For determination of phosphorylated proteins, 5 *μ*g whole cell lysate from serum-starved cells was resolved by SDS–PAGE and electroblotted onto nitrocellulose membranes. After transfer and blocking in 5% nonfat milk, primary antibody staining was carried out by incubation overnight at 4°C, and secondary antibody staining was for 1 h at room temperature. The chemiluminescence detection method (ECL) was used for all Western blot experiments.

### Crosslinking assay

Crosslinking of receptors were carried out as described (Montgomery, 2002). Briefly, gefitinib-treated cells were washed twice in ice-cold phosphate-buffered saline (PBS) and solubilised in RIPA buffer containing protease and phosphatase inhibitors, 10% glycerol and 1 mM bis(sulfosuccinimidyl) suberate (BS^3^) for 20 min at 4°C. Glycine at a final concentration of 250 mM was subsequently added for 5 min, followed by centrifugation at 14 000 **g** for 10 min. Equivalent amounts of protein were resolved by SDS–PAGE and electroblotted onto nitrocellulose membranes. Blotting and antibody incubations were performed as above using anti-EGFR and antiphospho-tyrosine antibodies.

### Proliferation assay

Exponentially growing cells were seeded in sextuple in 96-well plates at a concentration of 2000 cells/well, allowed to adhere and subsequently washed in PBS and incubated overnight in medium containing 0.5% FCS. Cells were then treated with varying concentrations of Iressa or the solute control DMSO and EGF. The optimal EGF concentration for inducing proliferation of NR6wtEGFR and NR6W cells has previously been determined and hence NR6wtEGFR and NR6W cells were added 10 and 0.1 nM EGF, respectively (Pedersen *et al*, unpublished observation). NR6 and NR6M cells were not added EGF. After 72 h the amount of cells were measured by performing a 3-[4,5-dimethylthiazol-2-yl]-2,5-diphenyltetrazolium bromide (MTT) proliferation assay (Roche, Denmark) (Hansen *et al*, 2003).

### Soft agar assay for anchorage-independent growth

Exponentially growing cells (1 × 10^5^) were suspended in 3 ml 0.5% (w/v) NuSieve low-melting agar (FMC, Rockland, ME, USA) dissolved in DMEM+0.5% FCS and plated in six-well plates covered with 0.5% agar dissolved in DMEM+0.5% FCS. Cells were then treated with varying concentrations of Iressa or the solute control DMSO. The optimal concentration of EGF for inducing anchorage-independent growth of NR6wtEGFR and NR6W cells has previously been determined and hence NR6wtEGFR were added 10 and 0.1 nM EGF, respectively (Pedersen *et al*, unpublished observation). NR6 and NR6M were not stimulated with EGF. Cultures in triplicate for each condition were replenished with fresh medium once a week. After 3 weeks the plates were stained with crystal violet and colonies >50 cells were counted.

## RESULTS

### Expression of ErbB family members in the cell lines

**Figure 1:**
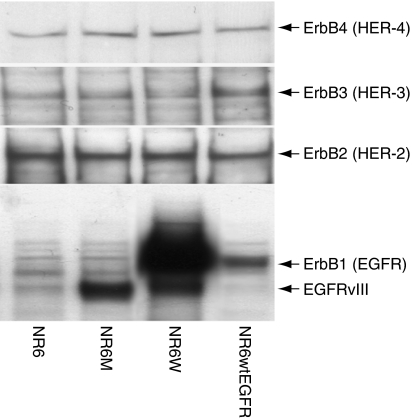
Expression of the ErbB family members in the cell lines described in these experiments as measured by immunoblotting using specific antibodies.

Epidermal growth factor receptor and EGFRvIII are known to form heterodimers with other members of the ErbB receptor family, which could potentially affect receptor phosphorylation and downstream signalling. Thus, the levels of ErbB1 (EGFR), ErbB2 (HER-2), ErbB3 (HER-3) and ErbB4 (HER-4) in the four NR6 cell lines: NR6, NR6wtEGFR, NR6W and NR6M were investigated by immunoblotting (Figure 1). All four cell lines have comparable levels of ErbB2, ErbB3 and ErbB4, which appear to be relatively low as compared to the levels of EGFR and EGFRvIII. Consequently, heterodimerisation most likely play a minor role in these cell lines.

### Effect of gefitinib on inhibition of EGFR tyrosine phosphorylations

**Figure 2:**
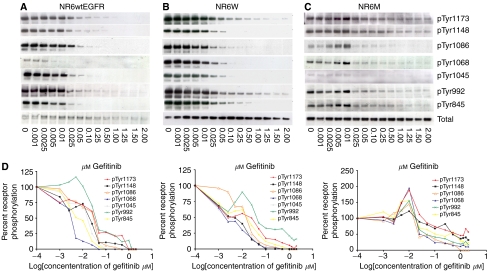
Profiles of EGF- (10 nM for 10 min) induced phosphorylation of various tyrosine residues of EGFR in the presence of varying concentrations of gefitinib in (**A**) the low-EGFR-expressing cell line NR6wtEGFR and (**B**) the high-EGFR-expressing cell line NR6W. (**C**) Profiles of constitutive phosphorylation of various tyrosine residues of EGFRvIII in the presence of varying concentrations of gefitinib. The phosphorylations were determined by immunoblotting of whole cell lysates with antibodies that specifically recognise the phosphorylated amino-acid residues Tyr845, Tyr992, Tyr1045, Tyr1068, Tyr1086, Tyr1148 and Tyr1173. Total EGFR or EGFRvIII levels are also indicated. (**D**) Bands were quantified using Kodak Digital Science Software version 1.0 corrected for total receptor level and plotted as a percentage of the phosphorylation levels in the untreated cells (lower panel). Exposures of the various blots are optimised for quantitative detection of IC_50_ and thus not directly comparable.

To investigate the efficacy of gefitinib towards inhibition of wt and mutant EGF receptors, we evaluated the effect of varying concentrations (0.001–2 *μ*M) of the inhibitor on receptor phosphorylations in the cell lines: NR6wtEGFR, NR6W and NR6M by immunoblotting (Figure 2). Initially cells were serum starved overnight to reduce baseline levels of phosphorylation. The cells were then mock treated (DMSO only) or treated with increasing concentrations of gefitinib for 5 h, after which they were stimulated with 10 nM of EGF for 10 min.

Immunoblot analysis of whole cell lysates revealed that in general gefitinib effectively inhibited all tyrosine phosphorylation sites on EGFR in both the high and low-EGFR-expressing cell lines (Figure 2A and B). However, the phosphorylation sites Tyr1173 and Tyr992 were less sensitive requiring higher concentrations of gefitinib for inhibition. The calculated IC_50_ values for these sites were 37 nM (Tyr1173), 37 nM (Tyr992), 26 nM (Tyr1173) and 57 nM (Tyr992) in, respectively, the low and high EGFR expressing cell lines (Table 1).

The tyrosine phosphorylation sites in the EGFRvIII-expressing cell line, although sensitive to gefitinib, were all very durable requiring 3–20 times higher doses for 50% inhibition as compared to the wt-EGFR (Figure 2C and Table 1). The Tyr1173 and Tyr1148 were the sturdiest sites with IC_50_ values of 97 and 214 nM, respectively. Thus, although EGFRvIII has a lower level of phosphorylation as compared to ligand-activated EGFR; the phosphotyrosine residues are more resistant to inhibition by gefitinib. Although an isolated increase in phosphorylation of EGFRvIII on some tyrosine residues was noted in the NR6M cells treated with 0.005 and 0.01 *μ*M (Figure 1C, lanes 4 and 5), this result was not consistently seen on repeat experiments.

The average IC_50_ values for tyrosine phosphorylation of EGFR in the low- and high-expressing cell lines were 22 and 21 nM, respectively, and thus independent of receptor expression levels. In contrast the average IC_50_ value of EGFRvIII tyrosine phosphorylation was 84 nM, suggesting that the EGFRvIII tyrosine kinase is approximately four times as resistant to gefitinib as EGFR (Table 1).

### Differential inhibition of intracellular signalling by gefitinib

The effect of gefitinib on the status of the major downstream targets of EGFR and EGFRvIII were also investigated. Extracellular regulated kinase-1 and -2 (ERK-2), phospholipase C *γ* (PLC-*γ*), protein kinase B (AKT) and the signal transducer and activator of transcription 3 (STAT3) are all essential downstream targets of EGFR and mediate many of its oncogenic effects.

**Figure 3:**
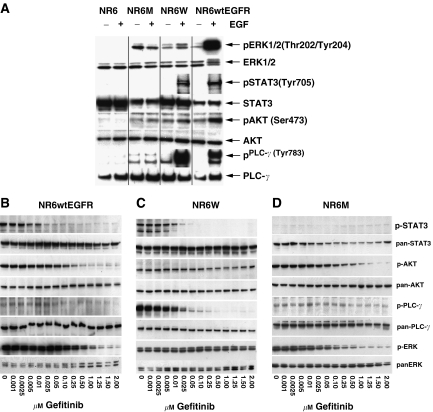
(**A**) Phosphorylation of key signalling molecules upon EGF (10 nM for 10 min) stimulation in the four cell lines. Profiles of EGF- (10 nM for 10 min) induced phosphorylation of various downstream signalling molecules of EGFR in cells pretreated with varying concentrations of gefitinib in (**B**) the low-EGFR-expressing cell line NR6wtEGFR and (**C**) in the high-EGFR-expressing cell line NR6W. (**D**) Profiles of EGFRvIII-induced phosphorylation of various downstream signalling molecules of EGFR in the presence of varying concentrations of gefitinib in the EGFRvIII-expressing cell line NR6M. The phosphorylations and total levels of the signalling molecules were determined by immunoblotting of whole cell lysates with antibodies that specifically recognise the total and phosphorylated species of ERK, PLC-*γ*, AKT and STAT3.

EGF-induced phosphorylation of ERK in the low EGFR-expressing cell line, but not significantly in the high-EGFR-expressing cell line (Figure 3A). The EGFRvIII-expressing cell line had a low level of constitutively phosphorylated ERK as a result of the constitutively active receptor (Figure 3A). Using a concentration gradient it was found that higher concentrations of gefitinib were needed to inhibit ERK signalling (IC_50_ of 394 and 356 nM, respectively) as compared to the receptor phosphorylations (IC_50_ of 22 and 84 nM, respectively) in the NR6wtEGFR and NR6M cell lines (Table 2, Figure 3B and D). ERK phosphorylations in the NR6W cell line in contrast were unresponsive to gefitinib even at the highest concentrations indicating that the ERK activity in this cell line is not mediated by the EGFR (Figure 3C).

As opposed to the level of ERK phosphorylation the high EGFR-expressing cell line had a substantial level of PLC-*γ* phosphorylation after EGF stimulation as compared to the low-EGFR- and -EGFRvIII-expressing cell lines, respectively (Figure 3A). Gefitinib effectively blocked this phosphorylation with a calculated IC_50_ of 27 nM (Table 2 and Figure 3C). The NR6wtEGFR and NR6M cell lines had low levels of PLC-*γ* phosphorylations but the level in the NR6M cell line was more resistant to inhibition by gefitinib (IC_50_ of 43 and 369 nM, respectively) see Table 2, Figure 3B and D.

The levels of AKT phosphorylation are most considerable in the low-EGFR- and -EGFRvIII-expressing cell lines and unresponsive to EGF in the high-EGFR- and -EGFRvIII-expressing cell lines (Figure 3A). As was the case for ERK, gefitinib fails to effectively inhibit AKT phosphorylation in the high-EGFR-expressing cell line indicating that EGFR is not the major activator of AKT in this cell line. The low IC_50_ (7 nM), however show that the weak induction of AKT phosphorylation by EGFR in this cell line is efficiently blocked by gefitinib (Table 2). Gefitinib inhibits AKT phosphorylations, with IC_50_ values of 220 and 263 nM, in the low-EGFR- and –EGFRvIII-expressing cell lines, respectively (Table 2, Figure 3B, C and D). Only the wt receptor is able to activate STAT3 upon EGF stimulation of both the high- and low-EGFR-expressing cell lines (Figure 3A). Of the downstream targets of EGFR, STAT3 seems to be most dependent on high receptor phosphorylation. STAT3 phosphorylation is completely abolished at IC_50_ values of 17 and 8 nM, which is comparable to the levels needed for inhibition of receptor autophosphorylation (Table 2, Figure 3B and C). There is no effect of gefitinib on the very low background level of STAT3 phosphorylation in the EGFRvIII-expressing cell line.

### Inhibition of EGFRvIII driven cell proliferation requires higher concentrations of gefitinib than wt EGFR

**Figure 4:**
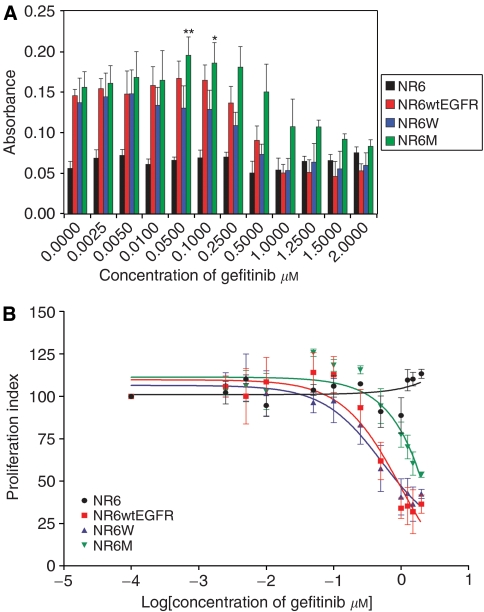
Effect of gefitinib on cell proliferation. (**A**) Graphical presentation of raw absorbance measurements for NR6, NR6wtEGFR, NR6W and NR6M cells treated with 0–2 *μ*M gefitinib for 72 h. NR6, NR6wtEGFR and NR6W cells were stimulated with EGF. This figure is representative of 15 independent experiments; bars, ±s.e. ^*^P<0.05 (compared to control treatment), ^**^P<0.01 (compared to control treatment). (**B**) Proliferation plotted as a percentage of the viability in the untreated cells; bars, ±s.e.

The rate of proliferation in the absence of gefitinib was higher in the EGFR- and EGFRvIII-expressing cell lines, as compared to the parental cell line NR6 (Figure 4A). Gefitinib had no effect on NR6 proliferation in the concentration range investigated. In contrast, proliferation of cells expressing EGFR decreased significantly, reaching a level similar to the parental cell line at a gefitinib concentration of 1 *μ*M. Proliferation of cell expressing EGFRvIII was more resistant to inhibition by gefitinib requiring a concentration of 2 *μ*M to reach a level comparable to that of the parental cell line (Figure 4A) The calculated IC_50_ values for inhibition of EGFR- and EGFRvIII-mediated proliferation were 0.97 and 0.52 *μ*M for the low- and high-EGFR-expressing cell lines, respectively, and 7.58 *μ*M for the EGFRvIII-expressing cell line (Figure 4B).

Notably, low levels of gefitinib (0.05–0.25 *μ*M) resulted in a slight, but statistically significant (*P*<0.05), increase in proliferation of EGFRvIII-expressing cells as compared to mock-treated cells (Figure 4A).

### Low concentrations of gefitinib increases the number of colonies of EGFRvIII-expressing cells

**Figure 5:**
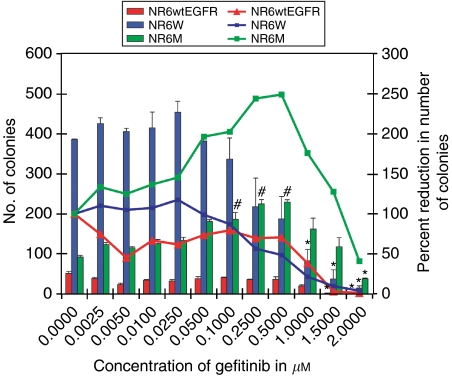
Growth of NR6 cells in soft agar in the presence and absence of gefitinib. (**A**) Graphical presentation of raw colony counts (left *y*-axis) and number of colonies plotted as a percentage of colonies in the untreated cells (right *y*-axis) for NR6, NR6wtEGFR, NR6W and NR6M cells treated with 0–2 *μ*M gefitinib and grown in soft agar for 18 days. NR6wtEGFR and NR6W were stimulated with 10 and 0.1 nM, respectively. This figure is representative of three independent experiments; bars, ±s.e. ^#^*P*<0.05 (increase as compared to control treatment), ^*^P<0.05 (decrease as compared to control treatment).

To investigate the effect of gefitinib on EGFRvIII-mediated transformation colony formation assays were performed in semisolid agar. Results confirmed that in the absence of gefitinib NR6W and NR6M cells readily form colonies in soft agar, whereas NR6wtEGFR cells require 10 nM EGF for weak colony formation (Figure 5). A gefitinib concentration above 0.1 *μ*M decreases the colony-forming ability of NR6W and NR6wtEGFR cells and a concentration of 1.5 *μ*M completely abolishes the ability of these cell lines to form colonies.

Surprisingly, gefitinib in the dose range from 0.1 to 0.5 *μ*M significantly facilitates, rather than abrogate, colony formation of NR6M cells. However, at a concentration of 2 *μ*M gefitinib completely blocks NR6M colony formation, as seen for the EGFR-expressing cell lines.

### Gefitinib-induced dimerisation of wt and mutant EGFR

**Figure 6:**
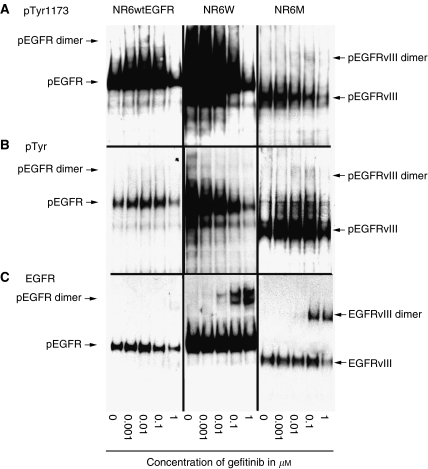
Dimerisation induction by gefitinib. Monolayer NR6wtEGFR, NR6W and NR6M cells were exposed to gefitinib for 24 h followed by exposure to the chemical crosslinker (BS^3^) followed by immunoblot analysis using (**A**) anti-EGFR(pTyr1173), (**B**) antiphospho-tyrosine (PY-20) and (**C**) anti-EGFR and antibodies.

As can be seen in Figure 6C gefitinib induces the formation of EGFR and EGFRvIII homodimers at concentrations of 0.01 *μ*M and above in the NR6W and NR6M cell lines. Furthermore, antiphosphotyrosine and antiphospho EGFR (Tyr1173) immunoblot analyses (Figure 6A and B) indicate that gefitinib in the concentration range 0.01–0.1 *μ*M increases the level of tyrosine-phosphorylated EGFRvIII monomers and homodimers. A higher gefitinib concentration (1 *μ*M) decreases the phosphotyrosine load of both monomers and dimers to levels lower than the untreated controls. In contrast, although gefitinib induces EGFR homodimerisation in the high-EGFR-expressing cell line, a strong decrease in phosphotyrosine load is found for both the monomer and dimer bands (Figure 6). This indicates that the induction of activated dimers by gefitinib only takes place in cells expressing EGFRvIII. Of note, the band with a molecular weight approximating EGFRvIII detected in the NR6W and NR6wtEGFR cell lines is not EGFRvIII as confirmed using RT–PCR and antibodies specific for EGFRvIII (data not shown). Rather, it possibly represents a degraded form of EGFR, the level of which depends on the lysis buffer, the primary antibody and the homogenisation procedure.

### Durability of gefitinib inhibition *in vitro*

**Figure 7:**
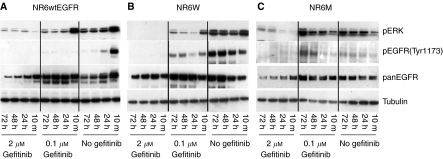
Duration of EGFR and EGFRvIII and ERK inhibition by gefitinib in the NR6wtEGFR, NR6W and NR6M cell lines. Profiles of EGFR and ERK phosphorylations in the presence of varying concentrations of gefitinib and at varying time points in (**A**) the low-EGFR-expressing cell line NR6wtEGFR and (**B**) the high-EGFR-expressing cell line NR6W both stimulated with 10 nM EGF. (**C**) Profiles of constitutive EGFRvIII phosphorylation and ERK in the presence of varying concentrations of gefitinib and at varying time points in the EGFRvIII-expressing cell line NR6M. The phosphorylations were determined by immunoblotting of whole cell lysates with antibodies that specifically recognise the phosphorylated species of ERK and EGFR-Tyr1173. Total levels of EGFR, EGFRvIII and tubulin are also shown.

A recent study has suggested that short-term treatment with gefitinib does not reduce phosphorylation of EGFRvIII and that repeated administration of gefitinib is necessary (Learn *et al*, 2004). Thus, to estimate the durability of gefitinib inhibition *in vitro*, an experiment was performed investigating the levels of EGFR/EGFRvIII and ERK phosphorylation in cells mock treated or treated with 0.1 or 2 mM of gefitinib and at the same time stimulated with 10 nM EGF for 10 min, 24, 48 and 72 h (Figure 7). The tyrosine phosphorylation site 1173 was selected, as it was the most durable of the phosphorylation sites. The results show that gefitinib rapidly and in a dose-dependent manner inhibits EGFR and ERK phosphorylations up to 72 h after EGF stimulation in both the high- and low-EGFR-expressing cell lines (Figure 7A and B). The decrease in EGFR levels, in the absence of gefitinib, in the low-EGFR-expressing cell line is due to receptor downregulation induced by EGF, a mechanism that appears not to be functioning in the NR6W and NR6M cell lines.

A concentration of 0.1 *μ*M gefitinib seems to increase EGFRvIII phosphorylation on residue 1173 and ERK phosphorylation after 48 and 72 h of treatment (Figure 7C). This could explain why gefitinib in low concentrations induce cell proliferation- and anchorage-independent growth. In contrast 2 *μ*M of gefitinib effectively inhibits both EGFRvIII and ERK phosphorylations in the NR6M cell line for up to 72 h. It is noteworthy that the high concentration of gefitinib seems to induce degradation of both EGFR and EGFRvIII independent of EGF. This is particularly evident in the low-EGFR-expressing cell line where 0.1 *μ*M gefitinib inhibit EGF-mediated downregulation, presumably due to its inhibition of the Tyr1045 site, but fails to do so at a concentration of 2 *μ*M (Figure 7A).

## DISCUSSION

### Phosphorylation of EGFR tyrosine residue 1173, AKT and ERK are least susceptible to inhibition by gefitinib

Gefitinib effectively inhibits phosphorylation of seven major tyrosine phosphorylation sites on EGFR and does so in cells expressing both low and high levels of EGFR. However, the effectiveness of inhibition of the individual sites varies with the Tyr1173 and Tyr992 being the more resistant in both cell lines. The levels of gefitinib sufficient to suppress EGFR phosphorylation are not sufficient to inhibit EGFRvIII phosphorylation. Average tyrosine phosphorylation of EGFRvIII being roughly four times more resistant to inhibition by gefitinib compared to the wt receptor with Tyr1148 and Tyr1173 being the more resistant sites. Thus, Tyr1173 appears to be the sturdiest tyrosine phosphorylation site in both EGFR and EGFRvIII requiring the highest concentration of gefitinib for inhibition. Tyr1173 is a major binding site for the adapter protein SHC and mediates activation of the RAS/RAF/MEK/ERK pathway (Batzer *et al*, 1994; Okabayashi *et al*, 1994). This is in concurrence with the observed persistence of ERK phosphorylation in the three cell lines even at high gefitinib concentrations.

As for ERK, the phosphorylation of AKT was resistant to gefitinib inhibition. The phosphorylation site(s) that mediates AKT phosphorylation is ill-defined, although Tyr1068 and Tyr1086 have been implicated through their binding to GRB2-associated binding protein 1 (GAB-1) (Rodrigues *et al*, 2000). As both Tyr1068 and Tyr1086 were sensitive to gefitinib inhibition it seems as if weakly phosphorylated receptors are able to facilitate AKT phosphorylation. The gefitinib-sensitive SRC (v-src sarcoma viral oncogene homolog) phosphorylation site Tyr845 on EGFR has been shown to mediate activation of STAT3, and indeed loss of EGF-dependent phosphorylation of STAT3 in response to gefitinib appears to be tightly coupled to suppression of EGFR autophosphorylation (Sato *et al*, 2003; Shao *et al*, 2003). Although EGFRvIII was phosphorylated on Tyr845, it was unable to induce STAT3 phosphorylation.

In general, the levels of gefitinib sufficient to inhibit the tyrosine phosphorylation sites on EGFR and EGFRvIII were insufficient to inhibit the downstream-signalling pathways, although phosphorylation of STAT3 was an exception. We believe that this is due to the amplification of the receptor signal seen by most signalling cascades (Mayawala *et al*, 2004). Targeting one or more kinases downstream of EGFR and EGFRvIII such as MEK, ERK or AKT could thus potentially increase the effectiveness of gefitinib. STAT3 is an exception as it binds directly to the receptor and following phosphorylation the protein translocates to the nucleus.

### Epidermal growth factor receptorvIII-mediated cell proliferation and anchorage-independent growth is both resistant and stimulated by gefitinib

Like EGFRvIII phosphorylation and signalling, EGFRvIII-driven proliferation and anchorage-independent growth was found to be less sensitive to inhibition by gefitinib compared to EGFR. However, the level of gefitinib required for inhibition of these cellular properties was much higher than those needed for inhibition of receptor phosphorylation. The relative instability of inhibitor over extended periods at room temperature may explain the discrepancy between gefitinib levels that were needed for 50% inhibition of proliferation (IC_50_ of 7.58 *μ*M) and colony formation (IC_50_ of 2 *μ*M) and those that were needed for 50% inhibition of autophosphorylation (average IC_50_ of 0.091 *μ*M).

Surprisingly, however, our results suggest not only that EGFRvIII-driven proliferation of NR6M cells is resistant to inhibition by gefitinib, but also appear to be enhanced by treatment with the inhibitor in the concentration range 0.05–0.25 *μ*M. A similar effect was observed for EGFRvIII-mediated anchorage-independent growth of NR6M cells, where colony formation was both resistant to and even stimulated by gefitinib in concentrations between 0.1 and 0.5 *μ*M.

Although a novel finding with regard to gefitinib, similar inductions of proliferation and colony formation of both EGFR- and EGFRvIII-expressing cells have been described in the literature with other quinazoline tyrosine kinase inhibitors (Montgomery, 2002; Li *et al*, 2003). Montgomery found that the EGFR-specific quinazoline inhibitor AG1478 at a concentration of 0.1 *μ*M stimulated colony formation of EGFRvIII-expressing cells (Montgomery, 2002). Similarly Li *et al* (2003) reported that PD153035 stimulated the proliferation of LN229/EGFR cells at 0.05 *μ*M. Thus, this seems to be a general effect of EGFR-specific quinazoline inhibitors, when used at certain concentrations.

The mechanism by which quinazolines increase proliferation and anchorage-independent growth of EGFRvIII-expressing cells is uncertain. We did not see an effect on phosphotyrosine load of and signalling by EGFRvIII after 5 h of gefitinib inhibition (Figures 2 and 3). However, upon prolonged exposure (>24 h) of EGFRvIII-expressing cells to gefitinib in the concentration range 0.01–0.1 *μ*M, EGFRvIII formed dimers and the phosphotyrosine load increased in both monomers and dimers. This is consistent with findings by Montgomery, who showed that AG1478 induced dimers of EGFRvIII in the concentration range 0.001-0.01 *μ*M, and that dimers similarly had increased phosphotyrosine load (Montgomery, 2002). Addition of higher concentrations of both gefitinib and AG1478 maintain the level of EGFRvIII dimers, but the phosphotyrosine load decreases accordingly. We speculate that EGFR-specific quinazoline inhibitors in certain concentrations, depending on its stability and the level of receptor, capture EGFRvIII in its dimer form. As the gefitinib concentration gradually decreases over time, it reaches a level where the receptor captured in the complexes is able to transphosphorylate the dimer partner. This is further supported by the observation that gefitinib in a concentration of 0.1 *μ*M initially (10 min–24 h) inhibited EGFRvIII phosphorylations on Tyr1173 and ERK, but after 48 h increased their phosphorylations (Figure 7). Higher concentrations (2 *μ*M) effectively blocked EGFRvIII and ERK phosphorylations for up to 72 h.

Although gefitinib was proficient at inducing dimers of EGFR in the high-EGFR-expressing cell line no increase in phosphotyrosine load could be detected. On the contrary gefitinib effectively inhibited phosphotyrosine load of both dimers and monomers.

Are the concentrations of gefitinib that stimulate EGFRvIII dimerisation, induce NR6M proliferation and anchorage-independent growth relevant in a clinical setting?

A recent study showed that daily oral dosing in the 400–600 mg day^−1^ range result in mean plasma concentrations ranging from 478 to 620 ng ml^−1^ (1.07–1.39 *μ*M) (Baselga *et al*, 2002). Assuming that the intratumour concentration equals the mean plasma concentration EGFRvIII phosphorylation and signalling should to be blocked, although cells are still proliferating at a higher rate than the control cells. However, if a lower daily dosing schema (225 mg day^−1^) is selected, the mean plasma concentration decreases to 160 ng ml^−1^ (0.36 *μ*M) (Baselga *et al*, 2002). At this concentration EGFRvIII is able to induce phosphorylation of both ERK- and AKT- and EGFRvIII-mediated cell proliferation is largely unaffected. However, it is possible that by maintaining a daily high dosing scheme, the unwanted stimulation of EGFRvIII by gefitinib may be avoided, and this warrants further investigations in the clinical setting.

In summary this study shows that gefitinib blocks EGFR- and EGFRvIII-mediated phosphorylation and signalling, although higher concentrations are needed for effective inhibition of EGFRvIII. A similar resistance to gefitinib was observed on EGFRvIII-driven proliferation and anchorage-independent growth. In addition, our data provide evidence that long-term exposure of EGFRvIII-expressing cells to low concentrations of gefitinib augment EGFRvIII phosphorylation, signalling, cellular proliferation and anchorage-independent growth. Further studies are needed to investigate the implications of these findings in a more clinical setting.

## Figures and Tables

**Table 1:** IC_50_ (nM) of specific EGFR tyrosine phosphorylation site inhibition by gefitinib

	**EGFR tyrosine phosphorylation sites**							
**Cells**	**Tyr1173**	**Tyr1148**	**Tyr1086**	**Tyr1068**	**Tyr1045**	**Tyr992**	**Tyr845**	**Average**
NR6wtEGFR	37	17	20	2	13	37	6	22
NR6W	26	5	12	3	4	57	18	21
NR6M	97	214	47	45	—	47	52	84

**Table 2:** IC_50_ (nM) of specific signalling molecule phosphorylation site inhibition by gefitinib

	**Inhibition of phosphorylation sites**				
**Cells**	**PLC-*γ***	**AKT**	**ERK**	**STAT3**	**Average**
NR6wtEGFR	43	220	394	17	169
NR6W	27	7	—	8	11
NR6M	369	263	356	—	329
